# A unified solution for different scenarios of predicting drug-target interactions via triple matrix factorization

**DOI:** 10.1186/s12918-018-0663-x

**Published:** 2018-12-31

**Authors:** Jian-Yu Shi, An-Qi Zhang, Shao-Wu Zhang, Kui-Tao Mao, Siu-Ming Yiu

**Affiliations:** 1 School of Life Sciences, Northwestern Polytechnical University, Xi’An, China; 2 School of Automations, Northwestern Polytechnical University, Xi’An, China; 3 School of Computer Science, Northwestern Polytechnical University, Xi’An, China; 4 Department of Computer Science, The University of Hong Kong, Hong Kong, China

**Keywords:** Drug-target interaction, Matrix factorization, Screening, Prediction, Cross-validation

## Abstract

**Background:**

During the identification of potential candidates, computational prediction of drug-target interactions (DTIs) is important to subsequent expensive validation in wet-lab. DTI screening considers four scenarios, depending on whether the drug is an existing or a new drug and whether the target is an existing or a new target. However, existing approaches have the following limitations. First, only a few of them can address the most difficult scenario (i.e., predicting interactions between new drugs and new targets). More importantly, none of the existing approaches could provide the explicit information for understanding the mechanism of forming interactions, such as the drug-target feature pairs contributing to the interactions.

**Results:**

In this paper, we propose a Triple Matrix Factorization-based model (TMF) to tackle these problems. Compared with former state-of-the-art predictive methods, TMF demonstrates its significant superiority by assessing the predictions on four benchmark datasets over four kinds of screening scenarios. Also, it exhibits its outperformance by validating predicted novel interactions. More importantly, by using PubChem fingerprints of chemical structures as drug features and occurring frequencies of amino acid trimer as protein features, TMF shows its ability to find out the features determining interactions, including dominant feature pairs, frequently occurring substructures, and conserved triplet of amino acids.

**Conclusions:**

Our TMF provides a unified framework of DTI prediction for all the screening scenarios. It also presents a new insight for the underlying mechanism of DTIs by indicating dominant features, which play important roles in the forming of DTI.

**Electronic supplementary material:**

The online version of this article (10.1186/s12918-018-0663-x) contains supplementary material, which is available to authorized users.

## Background

Identifying drug-target interactions (DTIs) is a crucial, but costly and time-consuming step in drug discovery, such as drug repositioning [1] and screening [2]. Computational methods (e.g. machine learning) play an important role to output interaction candidates for further validation in wet-lab experiments [1].

**Fig. 1:**
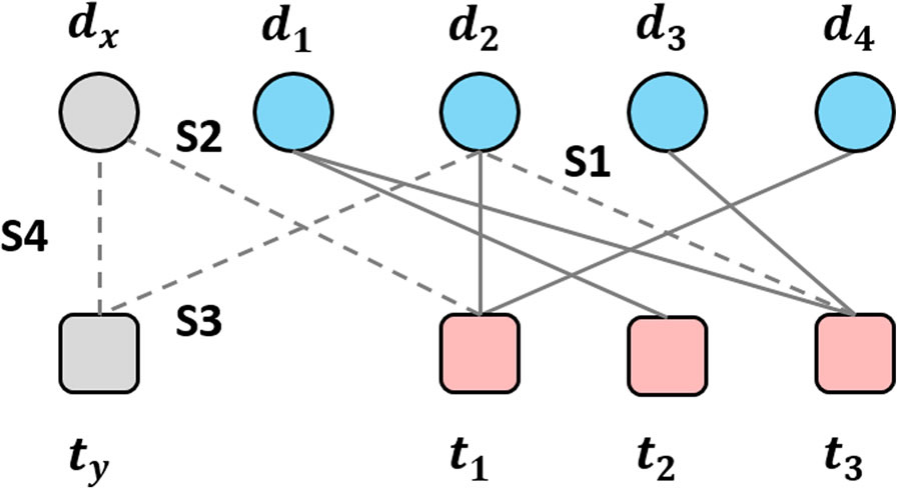
Illustration of four scenarios of screening DTIs. Circle nodes and rounded square nodes denote drugs and target respectively. Nodes d1, d2, d3 and d4 are known drugs and dx is a new chemical compound. Nodes t1, t2, and t3 are known targets and dy is the new protein. Solid lines linking nodes represent drug-target interactions. Dotted lines with labels indicate the scenarios

In general, there are four scenarios of screening DTIs [3], corresponding to drug repositioning, phenotypic screening, target-based screening as well as novel chemical compound-protein interaction prediction (Fig. 1). The first scenario (S1), predicting interactions between known drugs and known targets, accounts for drug-repositioning which tends to reuse or repurpose existing drugs on existing targets. The second scenarios (S2) accounts for testing new drugs on existing targets based phenotype approaches, while the third one (S3) accounts for applying existing drugs for a newly discovered target. The last scenario (S4), the most difficult case, accounts for screening the pairwise interacting candidates between newly discovered chemical compounds (drugs) and proteins (new targets).

Many, if not all, proposed computational approaches are based on machine learning. Their common fundamental assumption is that similar drugs tend to interact with similar targets. In terms of model type, the existing approaches can be roughly categorized into three groups: classification, network inference, and matrix factorization-based.

The classification-based models can further split into local classification model (LCM) and global classification model (GCM). For S2, by treating the drugs interacting and not interacting with a specific target as positives and negatives respectively, LCM builds a classifier to determine whether a given drug likely interacts with the target or not [4, 5]. LCM needs to build a set of separate classifiers for known targets. LCM usually requires different implementations for S3 and S1, of which the former is symmetric to S2 while the latter is the combination of S2 and S3. It cannot directly handle S4 because of no interaction to train in S4. More importantly, LCM cannot represent the relationship between the targets or the drugs (i.e., difficult to identify common or related features of the drugs (targets) that interact with the same target (drug)). Its extension provides an initial attempt to captures this relationship via the concept of a “super” operator (i.e., cluster the drugs/targets) [6]. In contrast, regarding the drug-target pairs having known interactions as positives and other pairs as negatives, GCM builds only one classifier, (such as [3, 5, 7–9]) based on the assumption that interactions and non-interactions are statistically separable and provides a “one-size-fits-all” approach for all predicting scenarios. However, GCM cannot represent the relationship between the targets or the drugs as well. Besides, the complexity of GCM is high because of tensor product-based similarity calculations or high-dimensional concatenate feature vectors. In general, the classification-based models are hardly able to capture the underlying structure among drug-target pairs (approved interactions and unknown pairs).

In fact, the interactions between drugs and targets are not independent, but show a significant relationship, which can be represented as a bi-partite network [10]. This network information derived from the essence of drug-target interactions could be utilized. Representing a set of DTIs as a bi-partite network, existing models based on network inference (e.g. NBI [11]) transform DTI prediction to link prediction between graph nodes. NBI utilizes two-step resource allocation to infer the potential links between nodes. However, it relies only on the local or the first-order topology of nodes and tend to completely bias to the high-degree nodes [9]. Besides, it cannot predict interactions for the cases of drug-target pairs without known reachable paths in the network, which is just one of intrinsic properties of DTI network containing isolated subnetworks [10]. These cases are come from S2, S3 and S4, and partially from S1. Heterogeneous network is a better promising model than the model based on resource allocation. Generally, a heterogeneous network is constructed by a DTI network and two additional networks generated by pairwise drug similarities and pairwise target similarities respectively. Random Walk with Restart was proposed to infer the potential links between drug nodes and target nodes [12, 13]. Nevertheless, existing methods based on heterogeneous network require seed nodes (both known drugs and known targets) which are hard to define appropriately in S4.

The models based on matrix factorization, such as BMF2K [14], CMF [15], NRLMF [16], provide an inspiring approach to capture the globally structural information between drug-target interactions. They project drugs and targets into a common low-rank feature space (usually called pharmacological space) according to drug similarity matrix and target similarity matrix. However, these models cannot explicitly indicate what features of drugs and targets significantly occur in interactions and non-interactions. Also, among them, only BMF2K can handle all four predicting scenarios. Neither CMF nor NRLMF can handle S4.

In drug design, pharmacologists are more concerned about the features that determinate or contribute to the interactions between drugs and targets. Especially, they prefer the pairwise features between drugs and targets in interactions, the shared features of drugs interacting with a common target and the shared features of targets interacting with common drugs. Some previous works have attempted to build the factor matrix to guide the prediction of drug-ligand interactions [17, 18], when 3D structures of targets are available. However, the availability is usually limited, especially for membrane proteins (e.g. GPCR and Ion Channel), which are the great majority of targets.

In this paper, we propose a triple matrix factorization (TMF) to capture the relationship between drug-target pairs in the latent pharmacological space. TMF enables us to build a unified solution of predicting DTI in all the four scenarios (Fig. 1). More importantly, it is able to indicate how often a pair of drug feature-target feature occurs in interactions or non-interactions, what the shared features of drugs interacting with a common target are, and what the shared features of targets interacting with a common drug are. The effectiveness of TMF is first demonstrated by comparing other state-of-the-art approaches on the benchmark of DTI datasets over both cross-validation in all the four scenarios and novel prediction, which deduces potential DTIs for drug repositioning. Then, another advantage of TMF is demonstrated by a case study, which identifies the common features of drugs sharing a target, the common features of target sharing a drug, as well as the crucial pairs between drug features and target features according to their occurrence in interactions and non-interactions.

## Methods

### Dataset

The DTI benchmark used in the following experiments was original constructed by Yamanishi et al. [19] and widely used in other sequential works [3, 7, 8, 14–16]. In terms of the types of targets in KEGG, it contains four datasets, including Enzymes (EN), Ion Channels (IC), G-Protein Coupled Receptors (GPCR), and Nuclear Receptors (NR). Table 1 shows their brief statistics. Each dataset contains three types of entries: the observed DTIs, the pairwise drug similarities, and the pairwise target similarities. They were organized into a DTI adjacent matrix, a drug similarity matrix and a target similarity matrix respectively and freely available at http://web.kuicr.kyoto-u.ac.jp/supp/yoshi/drugtarget/.

**Table 1:** Statistics of DTI benchmark datasets

	EN	IC	GPCR	NR
Number of drugs	445	210	223	54
Number of targets	664	204	95	26
Number of interactions	2926	1476	635	90

### Problem formulation

Given m drugs denoted as **D** = {*d*_1_, *d*_2_, …, *d*_*m*_}, n targets denoted as **T** = {*t*_1_, *t*_2_, …, *t*_*n*_}, and a set of interactions between them. These interactions are organized as the *m* × *n* DTI matrix denoted as **A**, in which *a*_*i*, *j*_ = 1 if drug *d*_*i*_ interacts with target *t*_*i*_ and *a*_*i*, *j*_ = 0 otherwise. Rows and columns in **A** are called as drug interaction profiles and target interaction profiles respectively. DTI matrix is also the adjacent matrix of DTI bipartite graph, so it can characterize the topological information between drug and target nodes in the graph. In addition, drugs or targets are usually characterized as highly-dimensional feature vectors (e.g. the fingerprints of drugs), or directly organized into a symmetric similarity matrix of which each entry is the pairwise drug similarity measured by algorithms (e.g. alignment). When given a similarity matrix, we can turn it into the corresponding feature matrix by singular value decomposition (see also Section Settings). Suppose that each drug can be represented a p-dimensional feature vector ({**d**_*i*_ ∈ **R**^*p*^, *i* = 1, 2, …, *m*}), and each target can be represented a q-dimensional feature vector ({**t**_*j*_ ∈ **R**^*q*^, *j* = 1, 2, …, *n*}). Therefore, the feature vectors of m drugs and n targets can be organized into the *m* × *p* feature matrix **F**_**d**_ and the *n* × *q* feature matrix **F**_**t**_ respectively.

We believe that drugs and targets can be mapped from their own feature spaces into a latent pharmacological space simultaneously and their inner products are correlated with their interactivity. The drug and the target in the pair corresponding to an interaction are near to each other in such a space, otherwise are far from each other. Thus, the DTI matrix can be represented as a triple matrix factorization (TMF),$\mathbf{A}\approx {\mathbf{F}}_d{\boldsymbol{\Theta} \mathbf{F}}_t^T$ where **Θ** is the bi-projection matrix, in which each entry indicates the importance of the pairs between drug features and target features among interactions and non-interactions. It builds the bridge between the features of drugs, the features of targets as well as the interactions between them.

Nevertheless, it cannot be directly solved by $\boldsymbol{\Theta} ={\left({\mathbf{F}}_{\mathbf{d}}\right)}^{-1}\mathbf{A}{\left({\mathbf{F}}_t^T\right)}^{-1}$ because of *p* ≫ *m* and *q* ≫ *n* in general. For example, drugs can be represented by an ordered list of binary bits (e.g. PubChem Fingerprint containing 881 bits), which characterize the substructures of drug chemical structure. Each bit represents a Boolean determination of the presence of an element (e.g. a type of ring system, SMART patterns) in a chemical structure [20]. By contrast, the number of drugs in the given benchmark dataset is possibly smaller the number of fingerprint bits. For instance, the biggest one (EN) in the benchmark datasets originally built by Yamanishi et al. [19] has only 445 drugs (See also Section A case study of interpreting dominant binding features). Some fingerprints, such as Klekota-Roth fingerprint having 4860 bits, may aggravate the difficulty of solving a regression model. Similar problem also arises in target features (e.g. K-mer having 20^K^ features).

Again, it cannot solved by $\boldsymbol{\Theta} ={\left({\mathbf{F}}_d^T{\mathbf{F}}_d\right)}^{-1}{\mathbf{F}}_d^T{\mathbf{AF}}_t{\left({\mathbf{F}}_t^T{\mathbf{F}}_t\right)}^{-1}$ as well because either ${\mathbf{F}}_d^T{\mathbf{F}}_d$ or ${\mathbf{F}}_t^T{\mathbf{F}}_t$could be nearly singular. The issue is usually caused by the multicollinearity among feature dimensions. Because the bits in drug fingerprint feature are ordered from simple to complex forms, there may have a dependency between bits. For example, the first four bits of PubChem Fingerprint indicate whether a chemical structure contains more than 4, 8, 16, and 32 hydrogen atoms respectively. Obviously, when the fourth bit is 1, the other three bits are surely 1 as well. Obviously, there is a multicollinearity among Fingerprint bit values.

As a result, we obtain **Θ** by solving the following optimization

1 $$ {\boldsymbol{\Theta}}^{\ast }=\arg \min \kern0.5em \left(J\left(\boldsymbol{\Theta} \right)={\left\Vert \mathbf{A}-{\mathbf{F}}_d{\boldsymbol{\Theta} \mathbf{F}}_t^T\right\Vert}_F^2+\lambda {\left\Vert \boldsymbol{\Theta} \right\Vert}_F^2\right). $$

Its solution can be achieved by Lagrange Multiplier Method. Let *∇J*(**Θ**) = 0, we can solve $-2{\mathbf{F}}_d^T{\mathbf{AF}}_t+2{\mathbf{F}}_d^T{\mathbf{F}}_d{\boldsymbol{\Theta} \mathbf{F}}_t^T{\mathbf{F}}_t+2\lambda \boldsymbol{\Theta} =0$ to obtain **Θ**^∗^. The equation is a form of Sylvester Equation: **AXB** + **CXD** = **E**, which can be rewritten as (**A** ⊗ **B** + **C** ⊗ **D**)*vet*(**X**) = *vet*(**E**), where *vet* is the stack of columns of matrix and ⊗ is Kronecker product. However, when both *p* and *q* are large numbers, Kronecker product generates a *pq* × *pq* matrix, which is too large to handle in memory.

Therefore, considering **A** is a low-rank matrix, we finally reformulate our problem by approximating it to another one as follows

$$ \left\{{\mathbf{A}}_d^{\ast },{\mathbf{A}}_t^{\ast },{\mathbf{B}}_d^{\ast },{\mathbf{B}}_t^{\ast}\right\}=\arg \min J\left({\mathbf{A}}_d,{\mathbf{A}}_t,{\mathbf{B}}_d,{\mathbf{B}}_t\right) $$

2 $$ J={\left\Vert \mathbf{A}-{\mathbf{A}}_d{\mathbf{A}}_t^T\right\Vert}_F^2+{\left\Vert {\mathbf{A}}_d-{\mathbf{F}}_d{\mathbf{B}}_d\right\Vert}_F^2+{\left\Vert {\mathbf{A}}_t-{\mathbf{F}}_t{\mathbf{B}}_t\right\Vert}_F^2+\lambda {\left\Vert {\mathbf{A}}_d\right\Vert}_F^2+\mu {\left\Vert {\mathbf{A}}_t\right\Vert}_F^2+\alpha {\left\Vert {\mathbf{B}}_d\right\Vert}_F^2+\beta {\left\Vert {\mathbf{B}}_t\right\Vert}_F^2 $$

where the first term in J denotes low-rank decomposition of **A**, the second denotes the linear regression between drugs’ latent interaction properties and their input features, the third term similarly accounts for the linear regression of targets, the last four terms are regularization terms. In detail, **A**_**d**_ is the *m* × *r* latent interacting matrix of drugs, **A**_**t**_ is the *n* × *r* latent interacting matrix of targets, and each row in **A**_**d**_ or **A**_**t**_ accounts for the latent topological properties of a drug or a target, because **A** can be treated as the adjacent matrix of DTI bipartite graph [10]. Their joint reflects the underlying pharmacological space. Moreover, **B**_**d**_ is the *p* × *r* regression coefficient matrix of drugs, **B**_**t**_ is the *q* × *r* regression coefficient matrix of targets, *r* ≤  *rank* (**A**), *α* and *β* are the positive coefficients for the regularization terms. Obviously, the number of entries/elements in the variables to be solved, (*m* + *n* + *p* + *q*) × *r*, in Formula (2) is fewer than that (*p* × *q*) in Formula (1) because usually *p* ≫ *m* and *q* ≫ *n*. Once both ${\mathbf{B}}_d^{\ast }$ and ${\mathbf{B}}_t^{\ast }$ are solved, **Θ**^∗^ can be easily achieved by ${\boldsymbol{\Theta}}^{\ast }={\mathbf{B}}_d^{\ast }{\left({\mathbf{B}}_t^{\ast}\right)}^T$.

The detailed solution can be achieved by Alternating Least Square, which iteratively solves a specific variable in turn by fixing other variables until reaching a convergence. In each round of its iterations, this procedure solves a set of equations {$\frac{\partial J}{\partial {\mathbf{A}}_d}=0$, $\frac{\partial J}{\partial {\mathbf{A}}_t}=0$, $\frac{\partial J}{\partial {\mathbf{B}}_d}=0$,$\frac{\partial J}{\partial {\mathbf{B}}_t}=0$} in turn, where the partial derivative functions are defined in Additional file 1. Since all the norms are Frobenius norm, their close-form solutions can be obtained as follows:

3 $$ {\displaystyle \begin{array}{l}{\mathbf{A}}_d=\left({\mathbf{A}\mathbf{A}}_t+{\mathbf{F}}_d{\mathbf{B}}_d\right){\left({\mathbf{A}}_t^T{\mathbf{A}}_t+\mathbf{I}+\lambda \mathbf{I}\right)}^{-1},\kern0.5em {\mathbf{A}}_t=\left({\mathbf{A}}^T{\mathbf{A}}_d+{\mathbf{F}}_t{\mathbf{B}}_t\right){\left({\mathbf{A}}_d^T{\mathbf{A}}_d+\mathbf{I}+\mu \mathbf{I}\right)}^{-1},\\ {}{\mathbf{B}}_d={\left({\mathbf{F}}_d^T{\mathbf{F}}_d+\alpha \mathbf{I}\right)}^{-1}{\mathbf{F}}_d^T{\mathbf{A}}_d,\kern0.5em {\mathbf{B}}_t={\left({\mathbf{F}}_t^T{\mathbf{F}}_t+\beta \mathbf{I}\right)}^{-1}{\mathbf{F}}_t^T{\mathbf{A}}_t.\end{array}} $$

Note that since some entries of A in S1 are unobserved, we cannot get the matrix-form solution involving A, but only the entry form of solution (See also Additional file 1).

### Unified predictive model

**Fig. 2:**
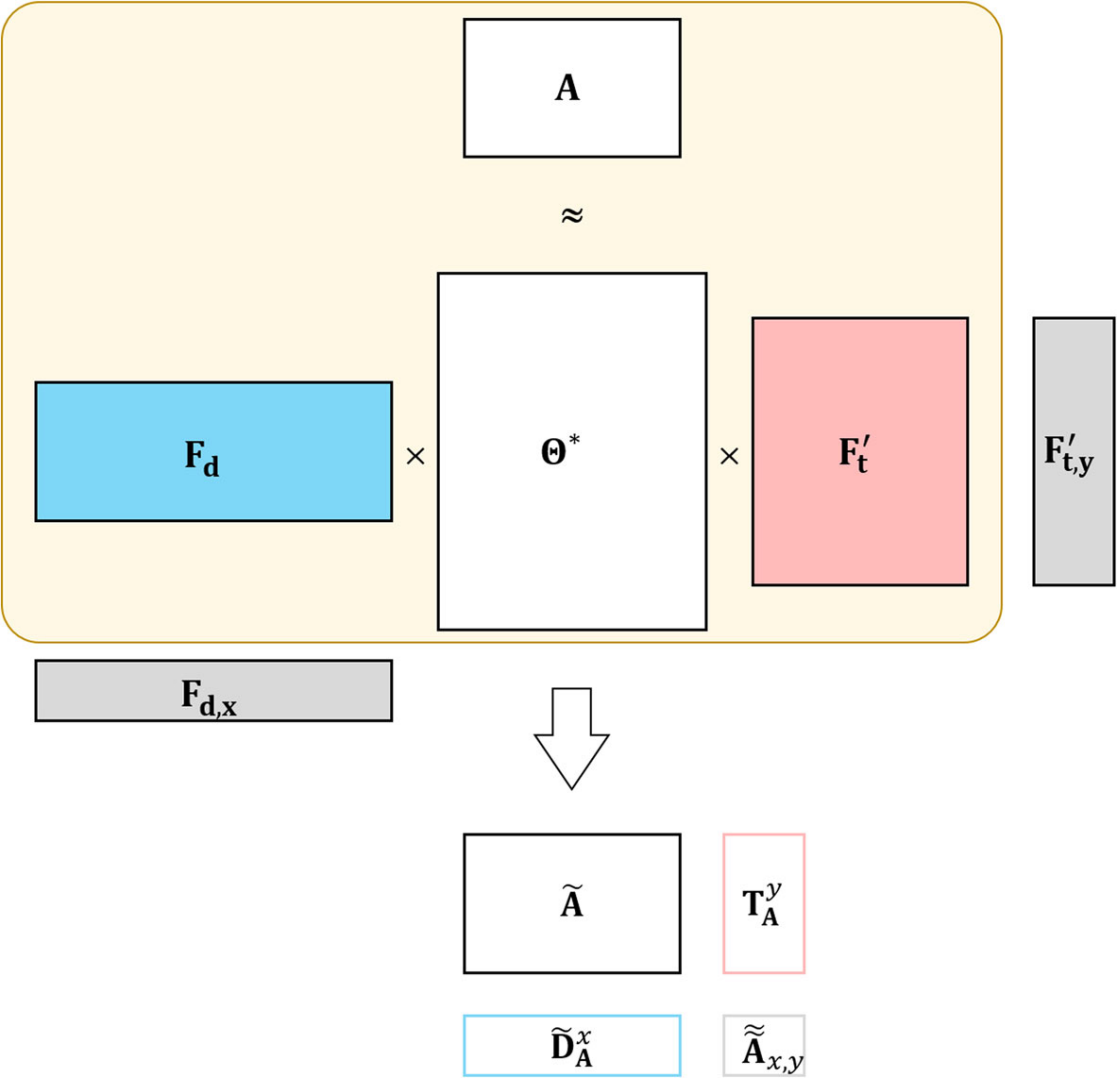
Illustration of triple matrix factorization over four predicting scenarios. The big rounded rectangle indicates the triple matrix factorization. **F**_**d**, *x*_ is the feature vector of the new drug *d*_*x*_ and **F**_**t**, *y*_ is the feature vector of the new protein *t*_*y*_. The predicted confidence scores for S1, S2, S3 and S4 are denoted by $\tilde{\mathbf{A}}$,${\tilde{\mathbf{D}}}_{\mathbf{A}}^x$,${\mathbf{T}}_{\mathbf{A}}^y$ and ${\tilde{\tilde{\mathbf{A}}}}_{x,y}$ respectively

After obtaining the bi-projection matrix **Θ**^∗^, we introduce a unified solution for the prediction in S1, S2, S3 and S4 based on the proposed bi-regression model (Fig. 2) as follows.

In the first scenario S1 (see also Fig. 1), our task is to infer how likely drug-target pairs are potential interactions. The confidence score of the testing entry **A**(*u*, *v*) in **A** is defined as,

4 $$ \tilde{\mathbf{A}}={\mathbf{F}}_{\mathbf{d},u}{\boldsymbol{\Theta}}^{\ast }{\mathbf{F}}_{\mathbf{t},v}^T $$

where **F**_**d**,*u*_ is the feature vector of *d*_*u*_ and **F**_**t**,*v*_ is the feature vector of *t*_*v*_

In S2, given a new drug *d*_*x*_, we aim to infer its interacting targets among **T**. Then the confidence score of *d*_*x*_ interacting with **T** is defined as

5 $$ {\tilde{\mathbf{D}}}_{\mathbf{A}}^x={\mathbf{F}}_{\mathbf{d},x}{\boldsymbol{\Theta}}^{\ast }{\mathbf{F}}_{\mathbf{t}}^T $$

where **F**_**d**,*x*_ is the feature vector of *d*_*x*_.

In S3, given a new target *t*_*y*_, we aim to infer its interacting targets among **D**. Then its confidence score of interacting with **D** is similarly defined as

6 $$ {\mathbf{T}}_{\mathbf{A}}^y={\mathbf{F}}_{\mathbf{d}}{\boldsymbol{\Theta}}^{\ast }{\mathbf{F}}_{\mathbf{t},y}^T $$

where **F**_**t**,*y*_ is the feature vector of *t*_*y*_.

In the most difficult scenario S4, our task is to find how likely a new drug *d*_*x*_ and a new target *t*_*y*_ interact with each other. The confidence score is defined as

7 $$ {\tilde{\tilde{\mathbf{A}}}}_{x,y}={\mathbf{F}}_{\mathbf{d},x}{\boldsymbol{\Theta}}^{\ast }{\mathbf{F}}_{\mathbf{t},y}^T $$

In practice, when *p* ≫ *m* or *q* ≫ *n*, we may calculate the confidence scores by ${\mathbf{F}}_{\mathbf{d}}{\mathbf{B}}_{\mathbf{d}}^{\ast }{\left({\mathbf{F}}_{\mathbf{t}}{\mathbf{B}}_{\mathbf{t}}^{\ast}\right)}^T$, but not directly by${\mathbf{F}}_{\mathbf{d}}{\boldsymbol{\Theta}}^{\ast }{\mathbf{F}}_{\mathbf{t}}^T$ since the size of **Θ**^∗^ is very large.

Obviously, TMF provides a unified form of solution for four types of DTI prediction by connecting drug feature space with the latent interaction topological space and connect target feature space with it simultaneously. This advantage would help achieve an inspiring DTI prediction but also provide an attempt to interpret why drugs interact with targets. Specifically, the regression coefficient matrix (**B**_**d**_ or **B**_**t**_) depicts the correlation between the feature matrix and the latent matrix. In other words, it is the bridge between the feature space and the interaction space. Consequently, we generate three significant matrices from **B**_**d**_ and/or **B**_**t**_ to investigate the DTI graph given in Fig. 1.

**The first one** is the *p* × *q* bi-projection matrix which is represented by ${\boldsymbol{\Theta}}^{\ast }={\mathbf{B}}_{\mathbf{d}}^{\ast }{\left({\mathbf{B}}_{\mathbf{t}}^{\ast}\right)}^T$. As its (i, j) entry can be positive, negative or zero, its sign indicates whether the pair of the i-th drug feature and the j-th target feature occur in interactions, non-interactions or not occur in all drug-target pairs, and its absolute value denotes the occurring intensity.

**The second one** is the *p* × *n* matrix ${\boldsymbol{\Theta}}_{\mathbf{d}}={\boldsymbol{\Theta}}^{\ast }{\mathbf{F}}_{\mathbf{t}}^T$called as Drug Projection Matrix, which maps the feature vectors of drugs (e.g. fingerprint) to their latent interaction topology (corresponding to S2). The sign of the (i, j) entry in **Θ**_**d**_ indicates: (1) the intensity of the i-th drug feature appearing in the set of drugs which interact with target j, if its value > 0; (2) the negative intensity of the i-th drug feature which doesn’t appear in the set of drugs interacting with target j, but appear in other drugs, when its value < 0; (3) no such drug feature appearing in all the drugs in the given dataset, if its value = 0.

**The third one** is the *m* × *q* matrix **Θ**_**t**_ = (**F**_**d**_**Θ**^∗^)^*T*^ called as Target Projection Matrix, which maps the feature vectors of targets (e.g. K-mer) to their interaction profiles (corresponding to S3). The entries in **Θ**_**t**_ having different signs also indicate significant meanings. The (i, j) entry represents (1) the intensity of the i-th target feature appearing in the set of targets which interact with drug j, if its value > 0; (2) the negative intensity of the i-th target feature not appearing in the set of targets which interact with drug j, but appearing in other targets, when its value < 0; (3) and no such feature appearing in all the targets in the given dataset, if its value = 0.

A case study of interpreting the abovementioned projection matrices shall be performed in Section *A Case Study of Interpreting Dominant Binding Features*.

### Cross validation and assessment

Remarkably, when assessing approaches, the appropriate schemes of cross validations for different scenarios should be adopted, otherwise over-optimistic results are perhaps obtained [3, 6, 21]. We generate different tasks of CV under four scenarios illustrated in Fig. 1 respectively:

S1: CV used the pairs between the drugs having >= 2 targets and the targets interacting with >= 2 drugs to avoid using the pairs, which should be used in three other scenarios. In each round of CV, some of these pairs are randomly selected for testing, and the union of the rest of them and other entries in A are used for training.

S2: CV is performed on drugs, where the rows corresponding to drugs in A are randomly blinded for testing and the resting rows are used for training.

S3: CV is performed on targets, where the columns in A (accounting for targets) are randomly blinded for testing and the resting columns are used for training.

S4: CV is performed on drug-target pairs, where the entries in A (drug-target pairs) are randomly selected for testing again, but all the rows and columns containing the testing entries are blinded for testing as well as training simultaneously. In other words, both the rows and the columns in A for training contain NONE of drugs or target involved in the testing entries.

**Fig. 3:**
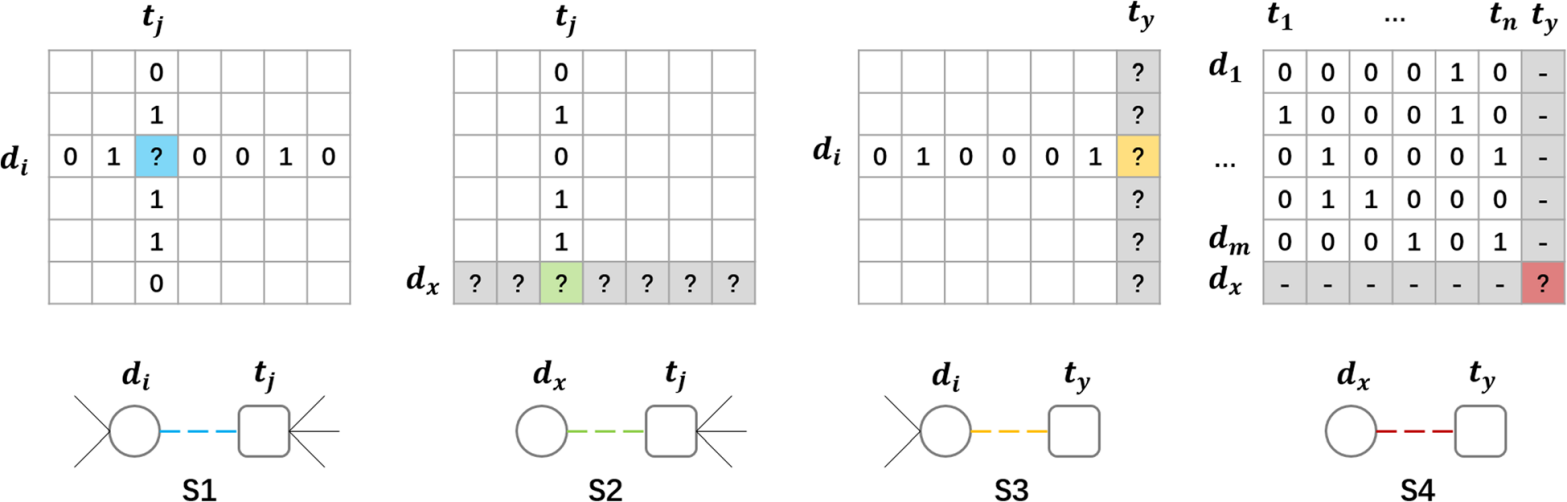
Illustration of cross-validation schemes for four scenarios. Each column accounts for a scenario. The first row contains the DTI matrices, in which the entries marked with “?” are the pairs of interest to be tested. Especially, the entries marked with “-” in S4 attend in neither training nor testing phases. The second row contains the topologies of the pairs of interest

In S1, S2 and S3, the same 10-CV as that in [16] is used. For example, in each round of S2, 90% of rows in Y are used as the training data and the remaining 10% of rows are used as the testing data. The similar procedures are adopted in both S1 and S3. Remarkably, because the CV of S4 is spanned by drug subsets and target subsets [3], a 10 × 10-CV in S4 contains 100 CV rounds, which would cause a large computation. Moreover, some rounds of the 10 × 10-CV could contain no positive drug-target pair in the testing set due to the sparse DTI network. This issue would cause a great variance over the CV when calculating precision and recall. Thus, considering the abovementioned distinctiveness of S4, we adopt a 5 × 5-CV. In detail, all the known drugs and all the known targets are randomly partitioned into five non-overlapping subsets of equal size respectively. In each round of the CV, each subset of drugs is removed as the testing drugs *Tst*_*d*_, each subset of targets is removed as the testing targets *Tst*_*t*_ and the remaining drugs and targets are severally referred to as the training drugs *Trn*_*d*_ and the training targets *Trn*_*t*_. All the entries between *Trn*_*d*_ and *Trn*_*t*_ in A are labelled as the training entries, only the entries between *Tst*_*d*_ and *Tst*_*t*_ in A are labelled as the testing entries, and the entries between *Tst*_*d*_ and *Trn*_*t*_ as well as those entries between *Trn*_*d*_ and *Tst*_*t*_ attend in neither training nor testing phases. An illustration of these CV schemes are shown in Fig. 3.

Former approaches usually use the Area Under the receiver operating characteristic Curve (AUC) to evaluate the performance of prediction. However, When the number of positive instances is much less than that of negative instances (e.g. DTI prediction), the area under precision-recall curve (AUPR) is more appropriate than AUC since it performs great penalty on highly-scored false positive predictions [15, 22]. Thus, we adopt AUPR to measure the performance of DTI prediction. The performance of DTI prediction is evaluated under K-fold cross-validation (K-CV) over N repetitions with different random seeds [16]. We calculate an AUPR score in each repetition of K-CV and report the average over N repetitions as the final AUPR score in the following experiments.

## Results and discussion

### Settings

Because the original datasets provide drug similarity matrices and target similarity matrices, we cannot direct utilize them. To accommodate both the drug similarity matrix and the target similarity matrix into our TMF, we applied singular value decomposition (SVD) to generate the corresponding feature matrices **F**_**d**_ and **F**_**t**_ by $S\overset{SVD}{=}{\mathbf{U}\boldsymbol{\Sigma } \mathbf{V}}^T=\mathbf{U}\sqrt{\boldsymbol{\Sigma}}\sqrt{{\boldsymbol{\Sigma}}^T}{\mathbf{V}}^T={\mathbf{FF}}^T$ before running TMF. Then, we set the starting point of four variables as follows: (1) considering that **A** is a non-full rank matrix and the equivalent and symmetric roles of **A**_**d**_ and **A**_**t**_, we generated the initial values of **A**_**d**_ and **A**_**t**_by SVD again by $\mathbf{A}\overset{SVD}{=}{\mathbf{U}}_{\mathbf{a}}{\boldsymbol{\Sigma}}_{\mathbf{a}}{\mathbf{V}}_{\mathbf{a}}^T={\mathbf{U}}_{\mathbf{a}}\sqrt{{\boldsymbol{\Sigma}}_{\mathbf{a}}}\sqrt{{\boldsymbol{\Sigma}}_{\mathbf{a}}^T}{\mathbf{V}}_{\mathbf{a}}^T={\mathbf{A}}_{\mathbf{d}}{\mathbf{A}}_{\mathbf{t}}^T$; (2) considering that both the number of features possibly greater than the number of drugs or targets and the multicollinearity among features, we utilize Partial Least-Squares Regression (PLSR) [23] to generate the initial values of **B**_**d**_ and **B**_**t**_ accordingly. Last, we set 10 as the number of fold in cross validation in the first three scenarios, 5 as that in the last scenario to guarantee the testing set in each round contains at least one positive instance, and 50 as the number of CV repetitions.

Moreover, we aim to demonstrate the superior ability of our TMF to find dominating pairs between drug features and target features, however, the features achieved by SVD on similarity matrices are latent features, which are not explicitly interpretable to pharmacologists. Therefore, we used PubChem fingerprints as drug features and the frequencies of amino acid trimers as target features. The former reflects the occurrence of chemical substructures, such as an element count, a type of ring system, atom pairing, atom nearest neighbors and SMARTS patterns. The latter characterizes the conservation of triple amino acids, which contribute to finding the binding pocks in proteins. The dominating feature pairs consisting of both important chemical structures and conserved protein sequence patterns are helpful to drug discovery, especially chemical structure design and binding pocket finding.

### Comparison with state-of-the-art approaches

Before running the comparison, we investigated how the dimension of the latent space influences the prediction. Taking NR dataset as an example, we tuned the value of *r* from the list {rank(A_trn_), rank(A_trn_)/2, rank(A_trn_)/3, rank(A_trn_)/4, rank(A_trn_)/5} by *λ* = *μ* = 1.0 and *α* = *β* = 0.5 in Scenario S1, where A_trn_ is the training adjacent matrix of DDI in each round of CV. Usually, the bigger the value of *r* is, the better the prediction is. Considering no significant improvement between in the first two cases of its values as well as the low-rank requirement, we chose the rank(A_trn_)/2 as its default value.

**Fig. 4:**
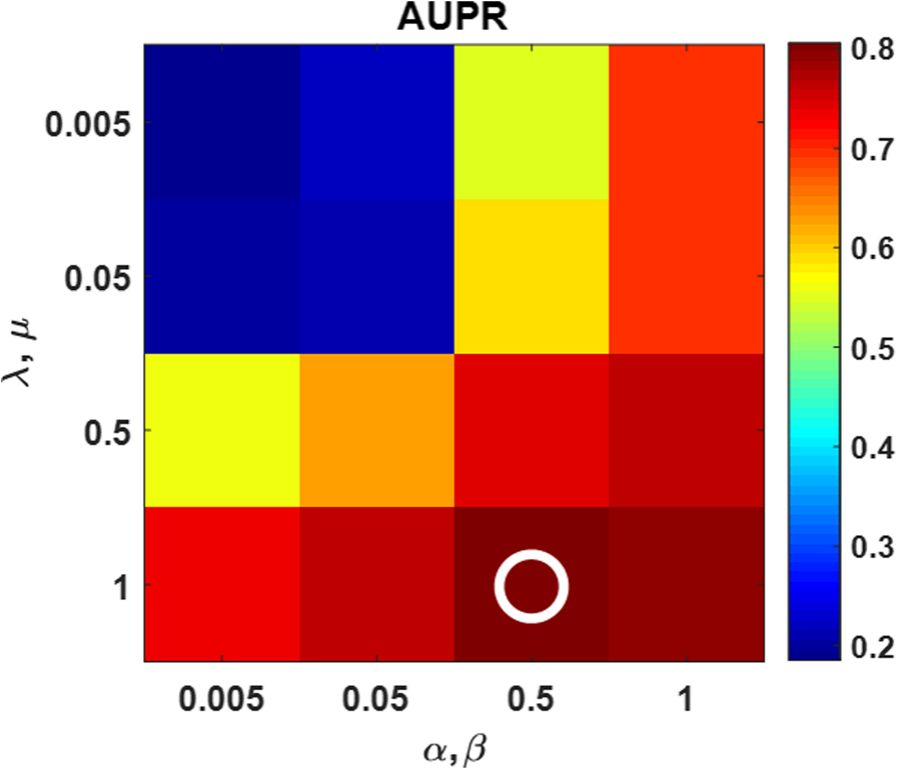
Example of how the regularization parameters influence the prediction on NR dataset in Scenario S1. The performance is also measured by AUPR. The best values of the parameters are *λ* = *μ* = 1.0 and *α* = *β* = 0.5, which are highlighted with a white circle

Moreover, we investigated how the four regularization parameters *λ*, *μ*, *α*, and *β* in Formula (2) influence the prediction under the condition of the latent dimension *r* = rank(A_trn_)/2. We tuned the values of *λ*, *μ*, *α*, and *β* from the list {0.005,0.05,0.5,1}. Considering the technically equal roles played by drugs and targets, we always set *λ* = *μ* and *α* = *β*. For example, the overview influence of tuning them on NR dataset in Scenario S1 is illustrated in Fig. 4.

In a similar way, after investigating all the scenarios across all the dataset, we finally determined the values of the four parameters as follows: for S1, *λ* = *μ* = 1.0 and *α* = *β* = 0.5; for S2, *λ* = *μ* = 0.05 and *α* = *β* = 0.5; for S3 *λ* = *μ* = 0.5 and *α* = *β* = 0.05; for S4 *λ* = *μ* = 0.05 and *α* = *β* = 0.5. Moreover, we found that the prediction is less sensitive to their values in the case of big datasets (e.g. EN, and IC) but more sensitive in the case of small datasets (e.g. NR) during the investigation. These values were used in the following experiments.

To validate the performance of TMF, we compared it with other state-of-the-art approaches, including NetLapRLS [7], WNN-GIP [8], RLScore [3], KBMF2K [14], CMF [15] and NRLMF [16], in both cross-validation and novel prediction.

During the cross validation, these approaches are able to cope with at least the first three scenarios. The set of the first three approaches exploits diverse classification-based models, while the set of the last three utilizes different matrix factorization-based models. Furthermore, we made an extra comparison with RLScore and KBMF2K in the last scenario because both of they can predict DTIs in this scenario. The comparison on four kinds of cross-validation schemes shows that our TMF is significantly superior to other approaches in terms of both AUPR (Table 2) and AUC (see Additional file 2).

**Table 2:** Comparison with state-of-the-art approaches in terms of AUPR

	NetLapRLS	WNN-GIP	RLScore	KBMF2K	CMF	NRLMF	TMF
S1-CV							
EN	0.789 ± 0.005	0.706 ± 0.017	0.828 ± 0.011	0.654 ± 0.008	0.877 ± 0.005	0.892 ± 0.006	**0.952 ± 0.002**
IC	0.837 ± 0.009	0.717 ± 0.020	0.769 ± 0.015	0.771 ± 0.009	0.923 ± 0.006	0.906 ± 0.008	**0.952 ± 0.002**
GPCR	0.616 ± 0.015	0.520 ± 0.021	0.625 ± 0.012	0.578 ± 0.018	0.745 ± 0.013	0.749 ± 0.015	**0.844 ± 0.006**
NR	0.465 ± 0.044	0.589 ± 0.034	0.526 ± 0.045	0.534 ± 0.050	0.584 ± 0.042	0.728 ± 0.041	**0.811 ± 0.035**
S2-CV							
EN	0.123 ± 0.009	0.278 ± 0.037	0.313 ± 0.031	0.263 ± 0.033	0.229 ± 0.020	0.358 ± 0.040	**0.438** ± **0.016**
IC	0.200 ± 0.026	0.258 ± 0.032	0.300 ± 0.020	0.308 ± 0.038	0.286 ± 0.030	0.344 ± 0.033	**0.376** ± **0.017**
GPCR	0.229 ± 0.017	0.295 ± 0.025	0.368 ± 0.025	0.366 ± 0.024	0.365 ± 0.022	0.364 ± 0.023	**0.428 ± 0.011**
NR	0.417 ± 0.048	0.504 ± 0.056	0.500 ± 0.058	0.477 ± 0.049	0.488 ± 0.050	**0.545 ± 0.054**	0.541 ± 0.033
S3-CV							
EN	0.669 ± 0.021	0.566 ± 0.038	0.794 ± 0.021	0.565 ± 0.023	0.698 ± 0.021	0.812 ± 0.018	**0.866 ± 0.007**
IC	0.737 ± 0.020	0.696 ± 0.035	0.781 ± 0.026	0.677 ± 0.021	0.620 ± 0.027	0.785 ± 0.028	**0.853 ± 0.008**
GPCR	0.334 ± 0.025	0.550 ± 0.047	0.533 ± 0.051	0.516 ± 0.045	0.433 ± 0.028	0.556 ± 0.038	**0.677 ± 0.028**
NR	0.449 ± 0.074	0.531 ± 0.073	0.433 ± 0.079	0.324 ± 0.071	0.400 ± 0.077	0.449 ± 0.079	**0.675 ± 0.062**
S4-CV							
EN	–	–	0.238 ± 0.018	0.211 ± 0.020	–	–	**0.265 ± 0.023**
IC	–	–	0.187 ± 0.020	0.232 ± 0.011	–	–	**0.251 ± 0.014**
GPCR	–	–	0.208 ± 0.017	0.111 ± 0.034	–	–	**0.231 ± 0.021**
NR	–	–	0.191 ± 0.051	0.231 ± 0.040	–	–	**0.239 ± 0.037**

In S1, S2, S3, the results generated by former approaches were reported by [16]. The best results in each benchmark dataset under four kinds of CVs are highlighted in bold face and the second-best results are underlined

Then, we evaluated TMF on predicting novel interactions, which are those highly-confident interactions not observed or labelled in the original benchmark datasets. Novel prediction reflects the ability of TMF on drug repositioning, which finds new uses for approved drugs. Unlike the ordinary cross validation, we deduce potential DTIs by the transductive inference, which uses the entire dataset as the training set and ranks the unknown drug-target pairs based on their interaction confidence scores generated by $\mathbf{A}={\mathbf{F}}_{\mathbf{d}}{\boldsymbol{\Theta}}^{\ast }{\mathbf{F}}_{\mathbf{t}}^T$. After ranking the unlabeled pairs with respect to their interaction scores, we picked up the top 10 predicted interactions as the interaction candidates. We further checked the predicted candidates in four popular databases, including DrugBank (D), KEGG (K), Matador (M) and ChEMBL (C), to validate the predicting performance of our model. An interaction candidate is marked with the first letter of database’s name if it is found in any of those databases (Table 3). The successful ratios of the number of top-10 validated candidates in four datasets are 70%, 90%, 90% and 50%. Compared with other approaches (Table 4), our model is able to achieve the best results of novel predictions across all the benchmark datasets with both larger average and less standard deviation of successful prediction ratios. These results demonstrate that our TMF is capable in both finding novel DTIs and helping preliminary screening of drugs in reality with the advantages of significant reduction of cost.

**Table 3:** De novo prediction on benchmark datasets

Rank	EN			IC			GPCR			NR		
1	D	D00947	hsa:4129	CD	D00546	hsa2566	K	D02250	hsa6751	C	D00182	hsa2099
2	M	D00528	hsa:1549	D	D00546	hsa2567	CD	D02358	hsa154	C	D00348	hsa6258
3	CMD	D00437	hsa:1559	CK	D00553	hsa6336	D	D00079	hsa5731	CK	D00348	hsa5915
4	–	D00188	hsa:1594	–	D05024	hsa774	KD	D00106	hsa5739	–	D00348	hsa190
5	M	D00437	hsa:1585	M	D00775	hsa2898	KD	D00095	hsa155	CKD	D00690	hsa2908
6	–	D03670	hsa:1579	CD	D00546	hsa2555	KD	D00442	hsa6755	–	D00348	hsa6096
7	D	D05458	hsa:4128	C	D01768	hsa6331	–	D00682	hsa5739	CK	D00348	hsa5916
8	D	D03365	hsa:1548	C	D00495	hsa8913	KD	D00095	hsa150	–	D00348	hsa6257
9	CD	D00097	hsa:5743	D	D00546	hsa2564	K	D00682	hsa5737	–	D00348	hsa6256
10	–	D00691	hsa:5152	CD	D00546	hsa2561	K	D00442	hsa6753	–	D00348	hsa6097

**Table 4:** Successful ratios of novel prediction among top-10 candidates

DB	NetLap-RLS	WNN-GIP	RLScore	KBMF-2 K	CMF	NRLMF	TMF
EN	70%	70%	70%	70%	20%	**90%**	70%
IC	60%	30%	50%	**100**%	0%	50%	90%
GPCR	40%	30%	60%	90%	50%	60%	**90%**
NR	10%	0%	20%	40%	10%	**50%**	**50%**
*Mean*	45%	33%	48%	**75%**	20%	63%	**75%**
*Std.*	0.265	0.287	0.263	0.265	0.216	0.189	0.191

The best results are highlighted in bold face. Mean and Std. denote the average of successful ratios and their standard deviation over four benchmark datasets respectively

To sum up, the superiority of TMF is validated by both cross-validations and novel prediction.

### A case study of interpreting dominant binding features

In this section, to dig out more factors determining interactions, we investigated the pairwise features between drugs and targets, the shared features of drugs interacting with a common target and the shared features of targets interacting with common drugs. Pharmacologists prefer interpretable drug/target features, however, the drug/target latent features generated from drug/target similarity matrix are uninterpretable. Thus, we adopted other explicit drug/target features to find dominating feature pairs contribute to form DTI.

Selecting NR dataset as the studying case, we applied PubChem fingerprint to characterize 2D structures of drugs and the frequencies of amino acid trimer (3-mer) to encode protein sequences of targets respectively. The PubChem fingerprint-based feature vectors of NR dataset is organized into the 54 × 881 feature matrix **F**_**d**_, while its trimer-based feature vectors is organized into the 26 × 8000 feature matrix **F**_**t**_ because the trimer contains 8000 (=20^3^) amino acid triplets.

PubChem fingerprint provides an ordered list of binary (1/0) bits, which indicate the occurrences of 881 specific substructures. They can be categorized into 7 groups, including Hierarchic Element Count (e.g. '>= 16 H' and '>= 32 C'), Ring in a canonic Extended Smallest Set of Smallest Rings(e.g. '>= 4 aromatic rings'), Simple Atom Pairs (e.g. 'Li-H' and 'C-S'), Simple Atom Nearest Neighbor(e.g. 'C(~C)(:C)(:N)'), Detailed Atom Neighborhood (e.g. 'C(#N)(-C)' and 'C(-C)(-C)(=O)'), Simple SMARTS Pattern (e.g. 'N#C-C=C' and 'N-C=C-[#1]') and Complex SMARTS Pattern (e.g. 'Cc1cc(S)ccc1'), where “~”, “:”, “-”, “=”, “#” match no bond order, bond aromaticity, single bond, double bond, and triple bond order respectively.

For targets, besides, we did not extract 3-mers on the whole sequences of targets (Nuclear Receptor proteins) in NR, but on the subsequences corresponding to their ligand-binding domains via the annotation in HGNC database [24], since all the proteins contain a DNA-binding domain and a ligand-binding domain.

To depict easily in the following sections, the pair of chemical substructure and amino acid triplet is referred as a feature pair.

#### Significant feature pairs

First, the bi-projection matrix **Θ**^∗^ since its (i, j) entry is able to reflect whether the pair of the i-th drug feature and the j-th target feature occur in interactions (positives), non-interactions (negatives) or not occur in all drug-target pairs (zeros).

By sorting all drug-target feature pairs their values, we first chose both the top positive feature pair {'C(#C)(-H)', 'GLR'} and the bottom negative feature pair {'C(~H)(~O)', 'LLL'} as two examples, to illustrate how frequently they appear in interactions and non-interactions respectively. After that, we counted the drugs out of 54 drugs and the targets out of 26 targets involved in the top/bottom pair, as well as the known interactions between them. Lastly, we measured how frequently the feature pair occurs in interactions by the ratio of the number of known involving interactions to the number of pairs between the drugs and the targets.

In detail, 6 drugs, 1 target and 5 interactions are involved in the top pair, while 31 drugs, 13 targets and only 28 interactions are involved in the bottom pair. For the top pair, the ratio of feature pairs attending interactions is 83.33% (=5/6). By contrast, for the bottom pair, the ratio of feature pairs attending non-interactions is 93.05% (=1–28/403). Similar results can be found in the top-n and the bottom-n pairs. Besides, all feature pairs having zero values represents their absence in the given drug-target pairs.

Consequently, the bi-projection matrix is able to indicate the feature pairs tending to occur in interactions, and the feature pairs tending to appear in the drug-target pairs of non-interactions. The greater the absolute values are, the stronger the tendency is. Meanwhile, it is also able to show that neither drug features nor target features in the zero-valued pairs are present among Nuclear Receptor and their drugs.

#### Frequently occurring substructures

Secondly, we investigated the *p* × *n* drug projection matrix ${\boldsymbol{\Theta}}_{\mathbf{d}}={\boldsymbol{\Theta}}^{\ast }{\mathbf{F}}_{\mathbf{t}}^T$, which can show at least two kinds of useful substructure patterns in PubChem fingerprint. One is the frequently occurring substructures FP1 of all drugs in NR dataset. Another is the significantly occurring and not occurring substructures FP2 of the drugs sharing a specific target.

To find out FP1, we counted the occurrence of each substructure having positive entries in **Θ**_**d**_. Those substructures are highly occurring (Table 5) in all the drugs of Nuclear Receptors. Consequently, FP1 may globally reveal a part of underlying common rules in designing drugs for Nuclear Receptors.

**Table 5:** Frequently occurring substructures(PubChem fingerprint) of the drugs in NR

Substructures	Group of PubChem fingerprint	Occurrence (> = 75%)
'C-C-C-C-C-C-C'	G6: Simple SMARTS pattern	0.8462
'C-C-C-C-C-C-C-C'	G6: Simple SMARTS pattern	0.8077
'C(-C)(-C)(=C)'	G5: Detailed atom neighborhood	0.8077
'> = 16 H'	G1: Hierarchic Element Count	0.8077
'Cc1cc(O)ccc1'	G7: Complex SMARTS pattern	0.7692
'C-N-C-[#1]'	G6: Simple SMARTS pattern	0.7692
'C(~H)(~N)'	G4: Simple atom nearest neighbor	0.7692
'> = 16 C'	G1: Hierarchic Element Count	0.7692

Each column in**Θ**_**d**_, accounting for a target, indicates how often chemical substructures (rows) appear in the drugs interacting with itself. Based on this, we can dig out the substructure patterns FP2. In details, target hsa7421, interacting with the drugs, D00129, D00187, D00188, D00299, and D00930, were selected as the example. After checking its top-4 substructures/fingerprints ('C-C=C-C=C', 'C=C-C=C', 'O-C-C-C=C' and '> = 32 H') in terms of substructure occurrence, we found that only these five drugs of “hsa7421” and two additional drugs ('D00211' and 'D01161') interacting with other targets contain all the four substructures. Meanwhile, after checking the bottom-2 substructures/fingerprints ('>= 3 any ring size 6' and 'Cc1ccc(C)cc1') which don’t occur in the drugs interacting with hsa7421, we found that both 'D00211' and 'D01161' contain all the two substructures. Consequently, FP2 is able to locally characterize the substructure occurrence of the drugs interacting with Target “hsa7421”. Meanwhile, it is able to differentiate these drugs of “hsa7421” from the drugs interacting with other targets which are different to “hsa7421”.

#### Conserved triplet of amino acids

Last, we analyzed the *m* × *q* target projection matrix ${\boldsymbol{\Theta}}_{\mathbf{t}}^T={\mathbf{F}}_{\mathbf{d}}{\boldsymbol{\Theta}}^{\ast }$. Similarly, it can also show at least two kinds of useful trimer patterns, including the common trimer patterns C1 of all targets in the dataset as well as the common trimer patterns C2 of the targets sharing a drug. These common patterns are potentially conserved.

To find out C1, we counted the occurrence of each amino acid triple having positive entries in ${\boldsymbol{\Theta}}_{\mathbf{t}}^T$ and picked up the most occurring triple. In NR dataset, it is ‘PGF’, which appears in the same position among 16 out of 26 targets, and of which its variants ‘PHF’, ‘PAF’, ‘PVF’, ‘PCF’, ‘SYF’, ‘DGF , ‘TGF’ and ‘SGF’ appear in the same position in the remaining target sequences. We also validated the conservation of this triplet pattern by the multiple sequence alignment tool, ClustalX (http://www.clustal.org/clustal2/) [25]. The alignment shows that ‘PGF’ and its variants are still matched in the same position without a gap. Thus, ‘PGF’ is the common trimer pattern in the given Nuclear Receptor proteins. Actually, it is just a part of the class-independent local motif (type 2) which is known as a ‘signature sequence’ for Nuclear Receptor [26].

In addition, each row denoting a drug in ${\boldsymbol{\Theta}}_{\mathbf{t}}^T$ corresponds to how amino acid triplets (columns) appear in the targets interacting with the drug. Based on it, the significance of C2 can be found. In details, two drugs D00577 (interacting with hsa2099, hsa2100, hsa2101, hsa2103, hsa2104) and D00585 (interacting with targets hsa2908, hsa367, hsa4306 and hsa5241) were selected as the example. According to the values of entries in ${\boldsymbol{\Theta}}_{\mathbf{t}}^T$, for D00577, the top-1 triplet is ‘LAD’ which is common in the sequences of its targets and is validated as a conserved trimer by ClustalX 2.1 as well. Other highly occurring triplets, such as ‘ALA’, ‘ELV’, show the similar results. For D00585, over 20 conserved triplets are found, including ‘QLT’, ‘RFY’, ‘QYS’, ‘FYQ’ and so on.

To summarize, the regression coefficient matrix is able to indicate how often a pair of drug feature-target feature occurs in interaction or non-interaction, what the shared features of drugs interacting with common targets are, and what the shared features of targets interacting with common drugs are.

## Conclusions

Computational approaches are able to predict candidates for screening DTIs. However, very most of them cannot be exploited in all the four scenarios of screening DTIs. Most importantly, none of them can explicitly indicate the features that determinate or contribute to DTIs. In this paper, through capturing the relationship between drug-target pairs in the pharmacological space, we have proposed TMF to address these issues. It is able to not only provide a unified solution to handle all the four scenarios of screening DTIs, but also to reveal the features of drugs and targets, which are critical for forming DTIs. Experimental results on the benchmark datasets have shown that TMF is significantly superior to existing state-of-the-art approaches in cross validations, and outperforms them in the novel prediction of DTIs by checking existing databases. More importantly, by revealing dominant features of DTIs, our TMF have provided an important insight for the underlying mechanism of DTIs. In addition, TMF can be applied in similar forms of problems in other areas, such as protein-protein interactions, drug-drug interactions [27–29], gene-disease associations, and non-coding RNA-disease associations [30], over not only binary but also real-valued relationship (i.e. binding affinity) between one kind of objects or two kinds of objects.

## Additional files

Additional file 1: Triple matrix factorization. (PDF 237 kb)

Additional file 2: **Table S1.** Supplementary comparison with state-of-the-art approaches in terms of AUC. (PDF 23 kb)

## Abbreviations

AUC: The area under the receiver operating characteristic curve
AUPR: The area under precision-recall curve
CV: Cross-validation
DTI: Drug-target interaction
GCM: Global classification model
LCM: Local classification model
TMF: Triple matrix factorization
