# Supplementary material for: A unified solution for different scenarios of predicting drug-target interactions via triple matrix factorization
Source: BMC Syst Biol. 2018 Dec 31;12(Suppl 9):136. doi: 10.1186/s12918-018-0663-x (PMC6311903; doi:10.1186/s12918-018-0663-x)
Supplement: Supplementary file 1 — Triple matrix factorization. (PDF 237 kb) [file 12918_2018_663_MOESM1_ESM.pdf]

Triple Matrix Factorization

Problem of Triple Matrix Factorization

Given the $m \times n$ DTI matrix denoted as $\mathbf{A}$, the $m \times p$ feature matrix as $\mathbf{F_d}$ and the $n \times q$ feature matrix as $\mathbf{F_t}$ respectively. Suppose that $\mathbf{A_d}$ is the $m \times r$ latent interacting matrix of drugs, $\mathbf{A_t}$ is the $n \times r$ latent interacting matrix of targets. Our task is to minimize the following objective function:

$$J = \| \mathbf{A} - \mathbf{A}_d \mathbf{A}_t^T \|_F^2 + \| \mathbf{A}_d - \mathbf{F}_d \mathbf{B}_d \|_F^2 + \| \mathbf{A}_t - \mathbf{F}_t \mathbf{B}_t \|_F^2 + \lambda \| \mathbf{A}_d \|_F^2 + \mu \| \mathbf{A}_t \|_F^2 + \alpha \| \mathbf{B}_d \|_F^2 + \beta \| \mathbf{B}_t \|_F^2 \quad (1)$$

Solution for S2, S3 and S4

The detailed solution can be achieved by Alternating Least Square (ALS), which iteratively solves a specific variable in turn by fixing other variables until reaching a convergence. In each round of its iterations, this procedure solves a set of equations in turn as follows $\{ \frac{\partial J}{\partial \mathbf{A}_d} = 0, \frac{\partial J}{\partial \mathbf{A}_t} = 0, \frac{\partial J}{\partial \mathbf{B}_d} = 0, \frac{\partial J}{\partial \mathbf{B}_t} = 0 \}$.

First, we solve the equations by the matrix-form formulas in the case of S2, S3 and S4 as follows:

$$\begin{aligned} &\frac{\partial J}{\partial \mathbf{A}_d} = 2\left(\mathbf{A} - \mathbf{A}_d \mathbf{A}_t^T\right)\left(-\mathbf{A}_t\right) + 2\left(\mathbf{A}_d - \mathbf{F}_d \mathbf{B}_d\right) + 2\lambda \mathbf{A}_d = 0 \\ &\Rightarrow \mathbf{A}_d = \left(\mathbf{A}\mathbf{A}_t + \mathbf{F}_d \mathbf{B}_d\right)\left(\mathbf{A}_t^T \mathbf{A}_t + \mathbf{I} + \lambda \mathbf{I}\right)^{-1} \end{aligned} \quad (2)$$

$$\begin{aligned} &\| \mathbf{A} - \mathbf{A}_d \mathbf{A}_t^T \|_F^2 = \| \mathbf{A}^T - \mathbf{A}_t \mathbf{A}_d^T \|_F^2 \\ &\frac{\partial J}{\partial \mathbf{A}_t} = 2\left(\mathbf{A}^T - \mathbf{A}_t \mathbf{A}_d^T\right)\left(-\mathbf{A}_d\right) + 2\left(\mathbf{A}_t - \mathbf{F}_t \mathbf{B}_t\right) + 2\mu \mathbf{A}_t = 0 \\ &\Rightarrow \mathbf{A}_t = \left(\mathbf{A}^T \mathbf{A}_d + \mathbf{F}_t \mathbf{B}_t\right)\left(\mathbf{A}_d^T \mathbf{A}_d + \mathbf{I} + \mu \mathbf{I}\right)^{-1} \end{aligned} \quad (3)$$

$$\begin{aligned} &\frac{\partial J}{\partial \mathbf{B}_d} = 2\left(-\mathbf{F}_d^T\right)\left(\mathbf{A}_d - \mathbf{F}_d \mathbf{B}_d\right) + 2\alpha \mathbf{B}_d = 0 \\ &\Rightarrow \mathbf{B}_d = \left(\mathbf{F}_d^T \mathbf{F}_d + \alpha \mathbf{I}\right)^{-1}\left(\mathbf{F}_d^T \mathbf{A}_d\right) \end{aligned}, \quad \begin{aligned} &\frac{\partial J}{\partial \mathbf{B}_t} = 2\left(-\mathbf{F}_t^T\right)\left(\mathbf{A}_t - \mathbf{F}_t \mathbf{B}_t\right) + 2\beta \mathbf{B}_t = 0 \\ &\Rightarrow \mathbf{B}_t = \left(\mathbf{F}_t^T \mathbf{F}_t + \beta \mathbf{I}\right)^{-1}\left(\mathbf{F}_t^T \mathbf{A}_t\right) \end{aligned}. \quad (4)$$

Solution for S1

Since some entries of A in S1 are unobserved, we cannot get the matrix-form solution involving A, but only the entry form of solution. To avoid the confusion of notions in the previous solution, we redefined the objective function:

$$
\begin{aligned}
J &= \sum_i \sum_j \left(a_{ij} - \sum_k u_{ik} v_{jk} \right)^2_{(i,j)\in\Omega} \\
&+ \sum_i \sum_k \left(u_{ik} - \sum_p f_{ip}^d b_{pk}^d \right)^2 + \sum_j \sum_k \left(v_{jk} - \sum_q f_{jq}^t b_{qk}^t \right)^2 \\
&+ \lambda \sum_i \sum_k (u_{ik})^2 + \mu \sum_j \sum_k (v_{ik})^2 \\
&+ \alpha \sum_i \sum_k (b_{pk}^d)^2 + \beta \sum_j \sum_k (b_{pk}^t)^2
\end{aligned} \quad , \qquad (5)
$$

where a_{ij} is the entry with the subscripts (i, j) in A, Ω denotes the set of the observed entries of A, u_{ik} , v_{ik} , f and b are the entries of $\mathbf{A}_d$, $\mathbf{A}_t$, feature matrices and regression coefficient matrices respectively. Thus, ALS can be used again to solve a set of equations in turn as follows { $\frac{\partial J}{\partial u_{ik}} = 0$, $\frac{\partial J}{\partial v_{jk}} = 0$, $\frac{\partial J}{\partial b_{pk}^d} = 0$, $\frac{\partial J}{\partial b_{pk}^t} = 0$ }. Because the last two equations are not coupled with A, we may still solve it by Formula (3). The solution of the first two equations for $(i, j) \in \Omega$ is as follows.

$$
\begin{aligned}
&\frac{\partial J}{\partial u_{ik}} = 2\left(\sum_j \left[\left(a_{ij} - \sum_s u_{is} v_{js} \right)(-v_{jk}) \right] + \left(u_{ik} - \sum_p f_{ip}^d b_{pk}^d \right) + \lambda u_{ik} \right) = 0 \\
&\Rightarrow u_{ik} = \frac{\sum_j \left[\left(a_{ij} - \sum_{s\neq k} u_{is} v_{js} \right) v_{jk} \right] + \sum_p f_{ip}^d b_{pk}^d}{(1+\lambda) + \sum_j (v_{jk})^2}
\end{aligned} \quad , \qquad (6)
$$

$$
\begin{aligned}
&\frac{\partial J}{\partial v_{jk}} = 2\left(\sum_j \left[\left(a_{ij} - \sum_t u_{it} v_{jt} \right)(-u_{ik}) \right] + \left(v_{jk} - \sum_q f_{jq}^t b_{qk}^t \right) + \mu v_{jk} \right) = 0 \\
&\Rightarrow v_{jk} = \frac{\sum_i \left[\left(a_{ij} - \sum_{t\neq k} u_{it} v_{jt} \right) u_{ik} \right] + \sum_q f_{jq}^t b_{qk}^t}{(1+\mu) + \sum_i (u_{ik})^2}
\end{aligned} \quad . \qquad (7)
$$
