# Supplementary material for: A unified solution for different scenarios of predicting drug-target interactions via triple matrix factorization
Source: BMC Syst Biol. 2018 Dec 31;12(Suppl 9):136. doi: 10.1186/s12918-018-0663-x (PMC6311903; doi:10.1186/s12918-018-0663-x)
Supplement: Supplementary file 2 — Table S1. Supplementary comparison with state-of-the-art approaches in terms of AUC. (PDF 23 kb) [file 12918_2018_663_MOESM2_ESM.pdf]

**Table a1.** Comparison with state-of-the-art approaches in terms of AUC

| | | NetLapRLS | WNN-GIP | RLScore | KBMF2K | CMF | NRLMF | TMF |
|---|---|---|---|---|---|---|---|---|
| S1 | EN | 0.972±0.002 | 0.964±0.003 | 0.962±0.003 | 0.905±0.003 | 0.969±0.002 | <u>0.987 ±0.001</u> | **0.989±0.001** |
| | IC | 0.969±0.003 | 0.959±0.003 | 0.967±0.003 | 0.961±0.003 | 0.981±0.002 | <u>0.989 ±0.001</u> | **0.989±0.001** |
| | GPCR | 0.915±0.006 | 0.944±0.005 | 0.925±0.007 | 0.926±0.006 | 0.940±0.007 | <u>0.969 ±0.004</u> | **0.983±0.003** |
| | NR | 0.850±0.021 | 0.901±0.017 | 0.850±0.030 | 0.877±0.023 | 0.864±0.026 | <u>0.950 ±0.011</u> | **0.978±0.008** |
| S2 | EN | 0.786±0.023 | **0.882±0.015** | 0.791±0.021 | 0.713±0.029 | 0.829±0.019 | <u>0.871±0.017</u> | 0.843±0.012 |
| | IC | 0.757±0.025 | 0.797±0.028 | 0.778±0.022 | 0.799±0.019 | 0.743±0.029 | <u>0.813 ±0.027</u> | **0.819±0.011** |
| | GPCR | 0.817±0.015 | 0.891±0.010 | 0.837±0.024 | 0.839±0.020 | 0.857±0.014 | **0.895±0.011** | 0.882±0.009 |
| | NR | 0.789±0.039 | <u>0.890±0.023</u> | 0.776±0.041 | 0.844±0.023 | 0.818±0.036 | **0.900±0.021** | 0.886±0.017 |
| S3 | EN | 0.905±0.014 | 0.947±0.008 | 0.931±0.009 | 0.876±0.012 | 0.915±0.013 | <u>0.966±0.005</u> | **0.976±0.001** |
| | IC | 0.914±0.012 | 0.950±0.007 | 0.937±0.009 | 0.938±0.008 | 0.905±0.012 | <u>0.964±0.007</u> | **0.972±0.002** |
| | GPCR | 0.770±0.02 | 0.926±0.013 | 0.853±0.017 | 0.882±0.016 | 0.837±0.019 | <u>0.930±0.012</u> | **0.959±0.007** |
| | NR | 0.655±0.046 | **0.935±0.017** | 0.736±0.034 | 0.668±0.060 | 0.680±0.066 | 0.851±0.027 | <u>0.929±0.029</u> |
| S4 | EN | - | - | 0.734±0.015 | <u>0.755±0.014</u> | - | - | **0.802±0.015** |
| | IC | - | - | 0.681±0.025 | <u>0.739±0.018</u> | - | - | **0.751±0.014** |
| | GPCR | - | - | <u>0.716±0.013</u> | 0.675±0.011 | - | - | **0.817±0.012** |
| | NR | - | - | 0.594±0.035 | <u>0.705±0.029</u> | - | - | **0.735±0.026** |

The best results in each benchmark dataset under four kinds of CVs are highlighted in bold face and the second-best results are underlined.
